# Draft genome of the fungus-growing termite pathogenic fungus *Ophiocordyceps bispora* (Ophiocordycipitaceae, Hypocreales, Ascomycota)

**DOI:** 10.1016/j.dib.2017.02.051

**Published:** 2017-03-08

**Authors:** Benjamin H. Conlon, Jannette Mitchell, Z. Wilhelm de Beer, Christian Carøe, M. Thomas P. Gilbert, Jørgen Eilenberg, Michael Poulsen, Henrik H. de Fine Licht

**Affiliations:** a Centre for Social Evolution, Section for Ecology and Evolution, Department of Biology, University of Copenhagen, Denmark; b Molecular Ecology, Institute of Biology, Martin-Luther-University Halle-Wittenberg, Hoher Weg 4, 06099 Halle an der Saale, Germany.; c ARC-PPRI Rietondale, 600 Soutpansberg Road, Rietondale, Pretoria, Gauteng, South Africa; ^d^Department of Microbiology and Plant Pathology, Forestry and Agriculture Biotechnology Institute, University of Pretoria, Pretoria, Gauteng 0001, South Africa; ^e^Centre for GeoGenetics, University of Copenhagen, Øster Voldgade 5-7, 1350 Copenhagen, Denmark; ^f^Section for Organismal Biology, Department of Plant and Environmental Sciences, University of Copenhagen, Thorvaldsensvej 40, 1871 Frederiksberg C, Denmark

## Abstract

This article documents the public availability of genome sequence data and assembled contigs representing the partial draft genome of *Ophiocordyceps bispora*. As one of the few known pathogens of fungus-farming termites, a draft genome of *O. bispora* represents the opportunity to further the understanding of disease and resistance in these complex termite societies. With the ongoing attempts to resolve the taxonomy of the Hypocralaean family, more genetic data will also help to shed light on the phylogenetic relationship between sexual and asexual life stages. Next generation sequence data is available from the European Nucleotide Archive (ENA) under accession PRJEB13655; run numbers: ERR1368522, ERR1368523, and ERR1368524. Genome assembly available from ENA under accession numbers: FKNF01000001–FKNF01000302. Gene prediction available as protein fasta, nucleotide fasta and GFF file from Mendeley Data with accession doi:10.17632/r99fd6g3s4.2 (http://dx.doi.org/10.17632/r99fd6g3s4.2).

**Specifications Table**

**Table:** 

Subject area	Biology
More specific subject area	Mycology, Genomics
Type of data	Genomic sequence, gene prediction and phylogenetic placement of *Ophiocordyceps bispora*
How data was acquired	Shotgun whole-genome DNA sequencing using the Illumina HiSeq platform at The Danish National High-Throughput DNA Sequencing Centre
Data format	Raw sequencing reads, Draft genome assembly, gene prediction and phylogenetic analysis
Experimental factors	DNA was extracted from *Ophiocordyceps bispora* fruiting bodies protruding from the thorax of two *Macrotermes* spp. alates.
Experimental features	The Geneious ver. 8.0.5. read-mapping and assembly tool, which also takes read quality into account when mapping, were used to assemble the draft *O. bispora* genome. The genome of the closely related *Hirsutella thompsonii* MTCC 6686 [1] was used as reference genome. Sequences used for phylogeny construction were identified using blastn searches.
Data source location	Samples were collected in 1993 from two alates of *Macrotermes* sp. with *O. bispora* fruiting bodies growing out from the thorax. The specimens were found in a small cavity excavated underneath a rock in Isiolo and Kajiado, Kenya.
Data accessibility	European Nucleotide Archive (BioProject: PRJEB13655; Runs: ERR1368522, ERR1368523, and ERR1368524; Contigs: FKNF01000001-FKNF01000302) and Mendeley Data (doi:10.17632/r99fd6g3s4.2, http://dx.doi.org/10.17632/r99fd6g3s4.2).

**Value of the data**

• *Ophiocordyceps bispora* is the first pathogen of fungus-farming termites (Macrotermitinae) to have its genome sequenced.
• *O. bispora* represents one of the few known parasites of fungus-farming social insects.
• The *O. bispora* draft genome assembly will be of value for future comparative and phylogenetic analyses.
• Further study of *O. bispora*׳s relationship with *Hirsutella thompsonii* could potentially identify a teleomorph–anamorph connection bringing the two species in line with the 2011 reccommendations for The International Code of Nomenclature for algae, fungi and plants [2].

## 1 Data

**Fig. 1:**
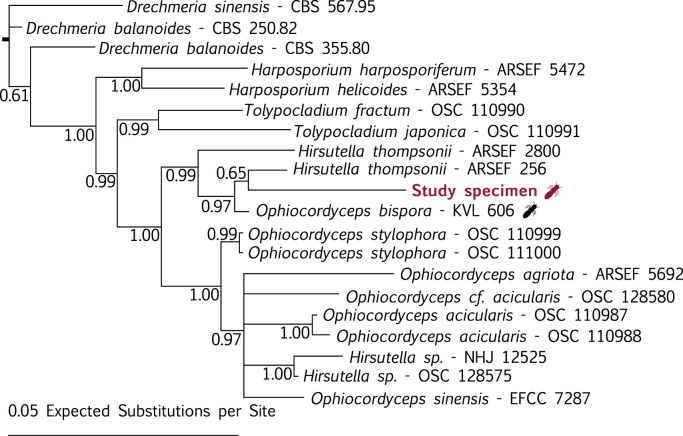
Phylogenetic placement of our specimen and closely related fungi in a concatenated nrSSU and nrLSU Bayesian phylogeny with posterior probabilities given at nodes. The sequenced specimen is highlighted in red while all other specimens are labelled with their herbarium numbers. A termite indicates that the fungus was reported from termites.

We present a partial draft genome assembly with gene prediction of the fungus-farming termite pathogenic fungus *Ophiocordyceps bispora* (Ophiocordycipitaceae, Hypocreales). *O. bispora* infects the reproductive alate caste of several termite genera, including fungus-growing termites in the genus *Macrotermes* in Africa [3], [4], [5], [6], [7]. It is one of the few known pathogens of fungus-farming social insects and its *Macrotermes* host apparently has no other known diseases [8], [9]. We also provide a two-gene phylogenetic analysis (Fig. 1) showing that *O. bispora* is closely related to an asexual form of *Ophiocordyceps*: *Hirsutella thompsonii*. The stark difference between sexual and asexual life stages in the Hypocrales family imply the different life stages of many species are classified twice [10]. Since 2011, the International Code of Nomenclature for algae, fungi and plants has been changed so that one fungus can have only one name [2]. Efforts to reconcile the taxonomy of the Hypocreales with these changes are ongoing [7], [11], [12], and the draft genome of *O. bispora* presented here may prove valuable in this effort.

## 2 Experimental design, materials and methods

### 2.1 Library

• Strategy: Shotgun whole-genome DNA sequencing.
• Taxon: *Ophiocordyceps bispora.*
• Sample details: Two alates of *Macrotermes* sp. with *O. bispora* fruiting bodies growing out from the thorax were collected from a small cavity excavated underneath a rock.
• Tissue: Fruiting body structures growing out from the dead, infected termite hosts.
• Location: Isiolo and Kajiado, Kenya.
• Sample handling: The sample was collected in 1993 by J. Eilenberg/G. Ochiel/H. Evans and preserved in ethanol at the Department of Plant and Environmental Sciences, University of Copenhagen.
• Selection: None.
• Layout: Single-end 100 bp reads.

### 2.2 Library construction protocol

Total fungal DNA was extracted from the fruiting bodies protruding from the termites by crushing and dissolving the sample in extraction buffer (10 mM Tris, 10 mM sodium chloride (NaCl), 5 mM calcium chloride (CaCl), 2.5 mM ethylenediaminetetraacetic acid (EDTA), 1% sodiumdodecyl sulfate (SDS), 10% proteinase K and 6.17 mg/mL dithiothreitol (DTT)) overnight at 56 °C, followed by organic extraction of the DNA using one volume of chloroform. The DNA-containing supernatant was purified by mixing with 10× volumes of modified PB buffer [13] and using a MinElute spin column (Qiagen) as demonstrated elsewhere [14]. The DNA was eluted in 60 μL EB buffer after a ten-minute incubation of the spin column with buffer at 37 °C and subsequently fragmented using a Diagenode Bioruptor with a program of 30 s on, 90 s off for 6 cycles. Illumina compatible shotgun sequencing library was generated using reagents from New England Biolabs kit for the 454, E#6070 L as described elsewhere [15]. The finished library was indexed and amplified for sequencing using 10 μL template and a mastermix consisting of 1× AmpliTaq Gold buffer (Invitrogen), 2.5 mM MgCl_2_ (Invitrogen), 0.8 μg/μL bovine serum albumin (BSA), 0.25 mM dNTP (Thermo Fischer Scientific, 25 mM stock), 0.2 μM forward and reverse indexed primer (10 μM stock), 0.2 U/μL AmpliTaq Gold enzyme (Invitrogen) and molecular grade water to a final reaction volume of 100 μL. The library was amplified in an Applied Biosystems 2720 Thermal Cycler, using the following conditions: 95 °C for 1 min, followed by a number of cycles of 95 °C for 30 sec, 60 °C for 30 s and 72 °C for 1 min for 5 cycles and finished by 7 min at 72 °C. Finally, the library was purified using a QiaQuick spin column (Qiagen), following manufacturer׳s instructions. The library was pooled with other samples and sequenced on 10% of a lane on an Illumina Hiseq 2500 instrument at The Danish National High-Throughput DNA Sequencing Centre, Copenhagen, Denmark.

### 2.3 Processing

Pipeline: DNA of the insect pathogenic fungus *O. bispora* was shotgun sequenced using Illumina HiSeq. Raw sequencing reads are available as three fastq files with accession nos. ERR1368522, ERR1368523, and ERR1368524.

• Run data file type: Fastq
• File Names (Runs: ERR1368522, ERR1368523, and ERR1368524, respectively):

○ TOG_QEHU_Term_fung_03_15_ACATAC_L004_R1_001.fastq.gz
○ TOG_QEHU_Term_fung_03_15_ACATAC_L004_R1_002.fastq.gz
○ TOG_QEHU_Term_fung_03_15_ACATAC_L004_R1_003.fastq.gz

The Geneious ver. 8.0.5. read-mapping and assembly tool, which also takes read quality into account when mapping, were used to assemble the draft *O. bispora* genome. The genome of the closely related *Hirsutella thompsonii* MTCC 6686 [1] was used as reference genome. The final assembled *O. bispora* draft genome consisted of 302 contigs and was 6,359,382 bp long. The maximum contig length, with a mean coverage of 3.1, was 158,451 bp with an N50 of 41,819 bp and a GC of 58.5%. Assembled contigs are available with accession nos. FKNF01000001-FKNF01000302. Protein coding sequences were predicted on both strands in the *O. bispora* draft genome using Augustus ver. 3.2.1 [16] using default parameters and found 3324 Open Reading Frames (ORFs).

The common fungal barcoding genes nuclear-ribosomal Large Sub-Unit (nrLSU) and nuclear ribosomal Small Sub-Unit (nrSSU) were located in the draft *O. bispora* genome assembly using blastn searches with Geneious 4.8.5. The identified *O. bispora* nrLSU and nrSSU sequences were combined with other Hypocrealean sequences from GenBank (Table 1), and a concatenated alignment of nrSSU and nrLSU was produced using MUSCLE [17]. A Bayesian phylogenetic analysis was performed using Topali v2 (Runs: 2; Generations: 1,500,000; Burn in: 50%) [18].

## Figures and Tables

**Table 1:** The fungal strains included in the phylogeny including known host taxa and GenBank accession numbers for nrLSU and nrSSU sequences.

Genus	Species	Strain	Host Taxa (where known)	nrLSU	nrSSU
*Drechmeria*	*balanoides*	CBS 250.82	Nematode	AF339539	AF339588
*Drechmeria*	*balanoides*	CBS 335.80	Nematode	AF339540	AF339589
*Drechmeria*	*sinensis*	CBS 567.95	Nematode	AF339545	AF339594
*Harposporium*	*helicoides*	ARSEF 5354	Nematode	AF339527	AF339577
*Harposporium*	*harposporiferum*	ARSEF 5472	Arthropod	AF339519	AF339569
*Hirsutella*	*sp.*	NHJ 12525	Hemiptera	EF469078	EF469125
*Hirsutella*	*sp.*	OSC 128575	Hemiptera	EF469079	EF469126
*Hirsutella*	*thompsonii*	ARSEF 256	Acari	KM652135	KM652090
*Hirsutella*	*thompsonii*	ARSEF 2800	Acari	KM652142	KM652095
*Ophiocordyceps*	*acicularis*	OSC 110987	Coleopteran larvae	EF468805	EF468950
*Ophiocordyceps*	*acicularis*	OSC 110988	Coleopteran larvae	EF468804	EF468951
*Ophiocordyceps*	*agriota*	ARSEF 5692	Coleoptera	DQ518754	DQ522540
*Ophiocordyceps*	*bispora*	KVL 606	Termite (Isoptera)	AF009654	AH006986
*Ophiocordyceps*	*cf.acicularis*	OSC 128580	Coleoptera	DQ518757	DQ588543
*Ophiocordyceps*	*sinensis*	EFCC 7287	Lepidopteran pupae	EF468827	EF468971
*Ophiocordyceps*	*stylophora*	OSC 110999	Coleopteran larvae	EF468837	EF468982
*Ophiocordyceps*	*stylophora*	OSC 111000	Coleopteran larvae	DQ518766	DQ522522
*Tolypocladium*	*fractum*	OSC 110990	Euteriomycete	DQ518759	DQ522545
*Tolypocladium*	*japonica*	OSC 110991	Euteriomycete	DQ518761	DQ588547
