# Supplementary material for: Draft genome of the fungus-growing termite pathogenic fungus Ophiocordyceps bispora (Ophiocordycipitaceae, Hypocreales, Ascomycota)
Source: Data Brief. 2017 Mar 8;11:537–42. doi: 10.1016/j.dib.2017.02.051 (PMC5357700; doi:10.1016/j.dib.2017.02.051)
Supplement: Supplementary file 1 — Supplementary material [file mmc1.docx]

**Confict of interest statement**

On behalf of all authors, I can confirm we have no competing conflicts of interest and all funding agencies are described in the manuscript.

Benjamin H. Conlon
